# Psychotherapy via videoconference: an Australian randomised controlled trial

**Published:** 2012-06-15

**Authors:** Daniel Robert Stubbings, Clare Rees, Lynne Roberts, Robert Kane

**Affiliations:** Curtin University of Technology, Australia; Curtin University of Technology, Australia; Curtin University of Technology, Australia; Curtin University of Technology, Australia

**Keywords:** telepsychology, videoconference, CBT, depression, anxiety

## Abstract

**Introduction:**

People who live in rural and remote regions often have little, if any, access to specialized mental health services. One way of addressing this issue is by providing such services via videoconference. Hence this study aimed to compare the effectiveness of psychotherapy provided in-person to via videoconference. This study is, to the author’s knowledge, the largest telepsychology adult project that has been conducted in Australia.

**Method:**

Twenty-nine clients were recruited who had a primary DSM-IV diagnosis on the depression-anxiety spectrum. Participants were randomly assigned to receive 12 sessions of either in-person or videoconference-based treatment. The intervention provided was based on cognitive-behavioural therapy (CBT) manualized treatments but individualized to suit the unique needs of each client. Primary symptomology and quality of life was measured before, after and 6 weeks following treatment. Secondary outcome measures included working alliance, credibility of therapy and client satisfaction.

**Results:**

Retention in both treatment conditions was similar. Statistical analysis using multi-level linear modeling indicated a significant reduction in client symptoms across time but no substantial differences between treatment conditions. There were also no substantial differences between conditions with regards to the working alliance, credibility of therapy and client satisfaction ratings.

**Conclusions:**

These findings suggest that CBT-based psychotherapy via videoconference can be effectively provided in a real-world clinical practice context.

